# Towards Better Vaccines: Enhancing Benefits, Mitigating Risks

**DOI:** 10.3390/vaccines14090809

**Published:** 2026-09-14

**Authors:** Kay Choong See

**Affiliations:** Division of Respiratory and Critical Care Medicine, Department of Medicine, National University Hospital, Singapore 119074, Singapore; kaychoongsee@nus.edu.sg

For any medical intervention, benefits must outweigh risks. Similarly, for vaccination to improve the lives of individuals and populations, vaccine efficacy (effect under controlled trial conditions) and effectiveness (effect under real-world contexts) must be proven, while side effects must be acceptable. These qualities cannot be assumed, even when vaccination has been commercially rolled out. For instance, the effectiveness of influenza vaccination wanes substantially over a single season [1]. In addition, post-licensure surveillance of a dengue vaccine identified excess cases of severe dengue in children who were vaccinated while seronegative [2]. These considerations underscore the importance of continually improving effectiveness and safety throughout vaccine development and implementation phases. In this Special Issue entitled “Clinical Strategies to Improve Efficacy, Effectiveness, and Safety of Vaccination in Humans,” 11 articles therefore addressed gaps in vaccine effectiveness and safety, including those unique to special populations.

The first group of four articles concern vaccine efficacy and effectiveness. Influenza vaccination currently relies on annual or semiannual administration of inactivated influenza vaccines targeting strain-specific viral surface glycoproteins [3]. These vaccines provide variable protection depending on how much vaccine strains match the circulating strains. To address this limitation, the OVX836 vaccine was developed to target viral nucleoprotein, which is better conserved across strains and could provide universal protection against influenza. Jacobs and colleagues then conducted a double-blind, placebo-controlled study of OVX836 in 100 older adults aged 65 years and above (https://doi.org/10.3390/vaccines12121391, accessed 30 August 2026). OVX836 generated significant antibody and cellular responses against a wide range of influenza strains. These encouraging results open the way for trials targeting clinically relevant endpoints such as reduction of symptomatic infection and severe complications of influenza. Like influenza vaccination, current COVID-19 vaccination relies on annual variant-adapted boosters [4]. Medeiros-Junior and colleagues identified a potential approach using half-dose boosters to extend vaccine coverage while conserving limited resources (https://doi.org/10.3390/vaccines13111113, accessed 30 August 2026). Although the adenoviral-vectored ChAdOx1 nCoV-19 vaccine is no more in use, their study involving 2801 adults aged 18–49 years showed that such a dose-sparing strategy maintains vaccine effectiveness and could be a model for other vaccines.

Respiratory syncytial virus (RSV) is increasingly recognized as a significant contributor of severe lower respiratory tract infections in older adults, with clinical consequences as severe as influenza and COVID-19 [5]. Commercially available vaccines target the prefusion conformation of the viral F protein, though other antigenic targets are also under study. Zhang and colleagues performed a double-blind randomized study of BARS13, an RSV G protein-based vaccine, among 125 healthy older adults aged 60–80 years (https://doi.org/10.3390/vaccines13080885, accessed 30 August 2026). They showed that BARS13 elicited strong immune responses with no serious adverse events, supporting further clinical development of G protein-based RSV vaccines. Separately, Cianci and colleagues reviewed the relationship between the host virome (the group of viruses existing in the human body [6]) and vaccine-induced immunity (https://doi.org/10.3390/vaccines13090895, accessed 30 August 2026). They highlighted emerging evidence suggesting that the host virome modulates vaccine immunogenicity and proposed that virome profiling could form part of a precision vaccine strategy, guiding both the intensity and timing of vaccination. 

The second group of four articles concern vaccine safety and adverse events. Widespread COVID-19 vaccination allowed the identification of rare but serious side effects, such as vaccine-induced immune thrombotic thrombocytopenia (VITT) [7]. VITT manifests as concurrent multiorgan thrombosis and severe thrombocytopenia. Sinischalchi and colleagues reviewed the mechanism of VITT, which involves generation of pathogenic antibodies against platelet factor 4 (https://doi.org/10.3390/vaccines14030225, accessed on 30 August 2026). Such insights inform both diagnosis and treatment approaches for this serious immunologic complication. Another potential side effect is antibody-dependent enhancement (ADE) of viral entry [8], where suboptimal levels of vaccine-induced antibodies paradoxically facilitate viral entry into host cells and increase disease severity. Although inactivated COVID-19 vaccines are not generally available now, Peng and colleagues studied 77 adults and demonstrated that inactivated vaccines generated lower levels of ADE-inducing antibodies compared to natural infection (https://doi.org/10.3390/vaccines13040425, accessed 30 August 2026).

Serious adverse events following vaccination may be uncommon, but surveillance in diverse settings remains important to maintain confidence in vaccine safety [9]. Pan and colleagues used the Chinese National Adverse Events Following Immunization (AEFI) Information System to show that pediatric *Haemophilus influenzae* Type b (Hib) vaccination in Zhejiang Province, China was associated with an AEFI rate of 63.01 per 100,000 doses from 2017 to 2023, with most being minor reactions like fever, injection site erythema, swelling and crying (https://doi.org/10.3390/vaccines13040349, accessed 30 August 2026). Among 1740 AEFIs, only six cases were severe (three febrile convulsions, two thrombocytopenic purpura, one allergic purpura), supporting Hib vaccine safety. Using similar methodology to support varicella vaccine safety, Liang and colleagues showed an AEFI rate of 34.79 per 100,000 doses, with 12 cases out of 1477 AEFIs categorized as severe (six thrombocytopenic purpura, two febrile seizures, one acute upper respiratory infection, one thrombocytopenia, one allergic purpura, one allergic rash) (https://doi.org/10.3390/vaccines13010057, accessed 30 August 2026).

The third and final group of three articles concern vaccination effectiveness and safety in special populations. Immunocompromised patients have an elevated risk of infection yet may mount a suboptimal immune response to vaccination [10]. Consequently, vaccine effectiveness and safety data from general populations may not be directly extrapolated to immunocompromised patients, necessitating dedicated studies in the latter. Kobayashi and colleagues showed that combination diphtheria, tetanus, and acellular pertussis vaccination in 29 adult allogeneic hematopoietic stem cell transplantation patients had good serological responses for several months (https://doi.org/10.3390/vaccines13030275, accessed 30 August 2026). However, serological responses sharply declined at one-year post-vaccination, highlighting the need for frequent boosters to maintain long term protection. Nonetheless, immunocompromised patients are a heterogenous group with varying degrees of immune suppression [10]. In contrast to Kobayashi’s results, Ake and colleagues showed that Ebola vaccination had good safety and sufficient immunogenicity in 25 people living with HIV compared to 50 people without HIV (https://doi.org/10.3390/vaccines12050497, accessed 30 August 2026). Separately, among 80 polio survivors, primary humoral immunodeficiencies might predispose unvaccinated patients to post-polio syndrome, a potentially debilitating disease marked by pain, weakness, fatigue, tiredness, and depressive symptoms [11]. Nevertheless, poliovirus neutralizing antibodies were high in these individuals, suggesting that polio vaccination remains a valuable strategy to prevent serious complications of natural infection in people with primary humoral immunodeficiencies (https://doi.org/10.3390/vaccines13090939, accessed 30 August 2026).

Collectively, authors of this Special Issue have highlighted a diverse range of topics and suggest that much work remains to be done in advancing vaccine effectiveness and safety. As such, we will be launching a second edition of this Special Issue to provide an additional avenue for more researchers to share important work aiming to make better vaccines for all.
